# Estimating Gender and Age from Brain Structural MRI of Children and Adolescents: A 3D Convolutional Neural Network Multitask Learning Model

**DOI:** 10.1155/2021/5550914

**Published:** 2021-05-26

**Authors:** Sergio Leonardo Mendes, Walter Hugo Lopez Pinaya, Pedro Pan, João Ricardo Sato

**Affiliations:** ^1^Center of Mathematics, Computing, and Cognition, Universidade Federal do ABC, Rua Arcturus no. 03, São Bernardo do Campo, SP 09606-070, Brazil; ^2^Department of Biomedical Engineering, King's College London, London SE1 7EH, UK; ^3^Escola Paulista de Medicina, Universidade Federal de São Paulo, R. Maj. Maragliano, 241 - Vila Mariana, São Paulo, SP 04017-030, Brazil

## Abstract

Despite recent advances, assessing biological measurements for neuropsychiatric disorders is still a challenge, where confounding variables such as gender and age (as a proxy for neurodevelopment) play an important role. This study explores brain structural magnetic resonance imaging (sMRI) from two public data sets (ABIDE-II and ADHD-200) with healthy control (HC, *N* = 894), autism spectrum disorder (ASD, *N* = 251), and attention deficit hyperactivity disorder (ADHD, *N* = 357) individuals. We used gray and white matter preprocessed via voxel-based morphometry (VBM) to train a 3D convolutional neural network with a multitask learning strategy to estimate gender, age, and mental health status from structural brain differences. Gradient-based methods were employed to generate attention maps, providing clinically relevant identification of most representative brain regions for models' decision-making. This approach resulted in satisfactory predictions for gender and age. ADHD-200-trained models, evaluated in 10-fold cross-validation procedures on test set, obtained a mean absolute error (MAE) of 1.43 years (±0.22 SD) for age prediction and an area under the curve (AUC) of 0.85 (±0.04 SD) for gender classification. In out-of-sample validation, the best-performing ADHD-200 models satisfactorily predicted age (MAE = 1.57 years) and gender (AUC = 0.89) in the ABIDE-II data set. The models' accuracy was in line with the current state-of-the-art machine learning applications in neuroimaging. Key regions for models' accuracy were presented as a meaningful graphical output. New implementations, such as the use of VBM along with a 3D convolutional neural network multitask learning model and a brain imaging graphical output, reinforce the relevance of the proposed workflow.

## 1. Introduction

One of the current challenges faced by the mental health research field is to include biological measurements for the assessment of psychiatry disorders [1, 2]. Despite recent advances [3], psychopathology remains mainly assessed through clinical interviews [4, 5]. Investigations on neuroimaging biomarkers, particularly in youth, may help clinicians in the hard task of differentiating typical from atypical developmental trajectories.

Among several potential biomarkers, structural magnetic resonance imaging (sMRI) is a promising method to enhance identification and precise classification in psychiatry [6–8]. Moreover, characterizing atypical brain structures from sMRI is an important step for understanding the mechanisms and etiology of these disorders to tailor treatments [9]. Over the past few decades, dozens of studies have identified brain structural changes in ASD and ADHD [9–11]. However, the vast majority of these findings are inconclusive, possibly due to methodological issues such as the use of small sample sizes, from a single study site, with little demographic variability (e.g., gender, age, or ethnicity) [9, 11]. These limitations have been recognized as a persistent source of bias in psychiatric classifications [12]. To achieve generalizable findings, one should employ large data samples, acquired from multiple sites/countries/scanners, including subjects with different ages, genders, ethnicities, and severity levels of psychiatry disorder [9, 11–13]. Fortunately, there are open data sets such as ABIDE-II and ADHD-200, which fit all these requirements.

Besides, most sMRI studies focused on traditional mass-univariate analytical methods, which are sensitive to gross and localized brain differences. These approaches, however, are not optimal for detecting subtle and spatially distributed neuroanatomical alterations, typically associated with psychiatric disorders [14, 15]. Therefore, machine learning techniques, such as deep learning networks, have shown interesting results in advancing group-level neuroimaging findings into individual-level clinically relevant classifications [16].

A specific deep learning network, called the convolutional neural network (CNN), revolutionized the computer vision area [17]. Regular CNNs use 2-dimensional images for their training process. This technical aspect, however, may cause loss of important data from the tridimensional (3D) structure of sMRI. A recent version of CNN, named CNN3D, overcomes this limitation by employing 3D images in its learning process, so it is an optimum candidate for sMRI applications. Recent studies, which used CNN to investigate psychopathologies, obtained better performance than the previously published literature [18–20]; however, none of these works employed a CNN3D trained with sMRI of youth to assess brain morphological features during neurodevelopment.

One downside of using deep learning models, such as CNN3D, is the low output interpretability, which sometimes provides little or no insight into the nature of the input data [14, 15]. To overcome this limitation, one can use a gradient-based algorithm such as SmoothGrad [21] to produce sensitivity voxel maps from input images that most contributed to models' decisions. Then, these attention maps can be intersected with a brain atlas such as AAL3 [22] to identify the top-focused brain regions of interest (ROIs) for the neural network decisions. This procedure may increase output interpretability and clinical relevance by showing brain ROIs with the greatest descriptive power for a given model prediction task. However, to date, few studies incorporated this approach. Moreover, integrating well-established sMRI processing techniques, such as voxel-based morphometry (VBM), into CNN3D training models seems to be appropriate to increase comparability to neuroimaging literature. VBM segments, aligns, and fits gray matter (GM) and white matter (WM) in a common spatial template, facilitating the hard task of comparing distinct clinical groups or gathering data for meta- or mega-analysis [23–26].

Different studies have contributed to the present knowledge on brain markers for psychiatric disorders, with several pieces of work assessing CNN3D [19, 20], multitask learning architecture [27, 28], and brain sMRI processed by VBM [9–11]. However, few studies have explored these methods jointly, particularly in large and heterogeneous data samples, to investigate biomarkers of neurodevelopment and psychiatric disorders across youth. The present study aims to evaluate a CNN3D model trained from ABIDE-II and ADHD-200 data sets to predict age (neurodevelopment), gender, and psychiatric disorder group (i.e., HC vs ASD or ADHD). We hypothesize that a CNN3D architecture, trained with 3D sMRI previously preprocessed by VBM, will detect complex patterns of morphological features in the human brain and allow correct classification of age, gender, and mental health status. Besides, we hypothesize that 3D saliency maps from trained models, generated via SmoothGrad [21], will provide identification of the brain's anatomical ROIs for each prediction task. These results could be intersected with 3D AAL3 brain atlas [22] and could be used to generate clinically relevant schematic representations of top-focused brain regions.

The current study evaluates the applicability of a workflow composed of carefully chosen methods and best practices to assess neurodevelopment from brain sMRI. First, the methods are described and justified in Section 2. Next, the achieved experimental results are presented in Section 3. Then, the results are discussed and compared to the related literature in Section 4. Finally, the conclusions are presented in Section 5.

## 2. Materials and Methods

### 2.1. Data Description

The data used in this study were obtained from two public data sets: Autism Brain Imaging Data Exchange II (ABIDE-II) and Attention Deficit Hyperactivity Disorder (ADHD-200). Both data sets can be downloaded from the NeuroImaging Tools & Resources Collaboratory Image Repository (NITRC-IR: https://www.nitrc.org/ir/). For this work, we used only one T1-weighted sMRI scan of each subject from the data sources. These images were collected from several locations in different countries: ABIDE-II includes 19 sites, and ADHD-200 includes 8 sites. Thus, the images' acquisition parameters vary due to different scanners' models and brands, ranging from 1.5T to 3T, each hosting a head coil from 8 to 32 channels. Detailed information and scanners' acquisition parameters can be retrieved from ABIDE-II (http://fcon_1000.projects.nitrc.org/indi/abide/abide_II.html) and ADHD-200 (http://fcon_1000.projects.nitrc.org/indi/adhd200/) documentation. The data were collected and made public according to the responsibility and approval of the given local ethics of each project.

### 2.2. Subjects

**Figure 1:**
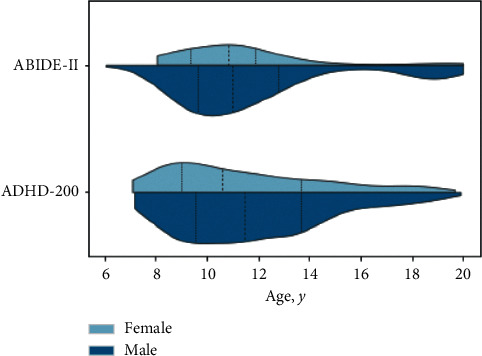
Subjects demographic distribution of ABIDE-II and ADHD-200 data sets. Vertical dotted lines show the quartiles. Ages are presented in years.

Since we focus on neurodevelopmental processes in children and adolescents, we discarded subjects older than 20 years of age. Some individuals had more than one sMRI scan in the data set (collected from different scanning sessions). In these cases, only the first sMRI of each subject was considered. Data without information on gender, age, and psychiatric disorder (i.e., HC, ASD, or ADHD) were also discarded. Furthermore, each subject belonged exclusively to ABIDE-II or ADHD-200 data set (no subject was in both). After applying these criteria, the sample for the present analysis and main demographic and phenotypic data are presented in Table 1 and Figure 1.

Individuals at different levels of the autism spectrum were grouped in the ASD label and, similarly, individuals with different subtypes of ADHD (inattention, hyperactivity, or combined) were grouped.

### 2.3. MRI Processing

The sMRI was processed using VBM [23] via the Statistical Parametric Mapping software (SPM12 v7771, from https://www.fil.ion.ucl.ac.uk/spm/software/). Briefly, VBM involves spatially normalizing all MRI images to the same stereotactic space, allowing extraction of different brain tissues from images partitioned with correction for nonuniform intensity variations [23]. In the past decades, VBM has been largely adopted in neuroimaging studies, such as the ones investigating ASD and ADHD [10]. The complete conceptual framework, methodology, and background behind the software are available in the Statistical Parametric Mapping book [29].

The data sets were processed using two batches of tasks (one batch for ABIDE-II and another for ADHD-200). Although the same procedures were applied to both data sets, we chose to process them in separate batches to ensure that each data set was completely independent. All the sMRI transformation steps were performed through the SPM12 software, following the VBM Tutorial [30].

First, sMRI data were spatially segmented to segregate GM and WM [24]. In this step, the skull, tissues, and artifacts outside the brain tissue are removed from the original image.

Second, the DARTEL algorithm [25] was applied to increase the accuracy of intersubject alignment. This transformation works by aligning GM among the images while simultaneously aligning WM during the generation of a template to which the data are iteratively aligned [26]. Third, the resulting files from the previous step were spatially normalized, Jacobian-scaled, and smoothed with a Gaussian full width at half maximum (FWHM) set to 8 mm to generate images in the Montreal Neurological Institute (MNI) coordinate system [31,32]. After these transformations, each sMRI scan produced two 3D matrices (one for GM and another for WM), with each voxel carrying the probable density of brain tissue at that location.

Finally, we loaded the previously transformed GM and WM via Python, through the SimpleITK library (https://simpleitk.org/) and applied a common mask assigning the value −1 to every background voxel (outside the brain). We chose to set the value −1 (instead of zero) to streamline the learning process of the models, due to the increase in the distance between background voxel values and brain voxel values with low tissue probability (close to zero). The brain matrices and their corresponding phenotypic data were saved in the TensorFlow record format (https://www.tensorflow.org/tutorials/load_data/tfrecord). This notation allows for better performance by storing data in binary linearly serialized files. As the data sets are still relatively large after the transformations (about 30 GB for both data sets), this step is important to read data efficiently during the model training phase.

### 2.4. Deep 3D Convolutional Neural Network Multitask Learning Architecture

**Figure 2:**
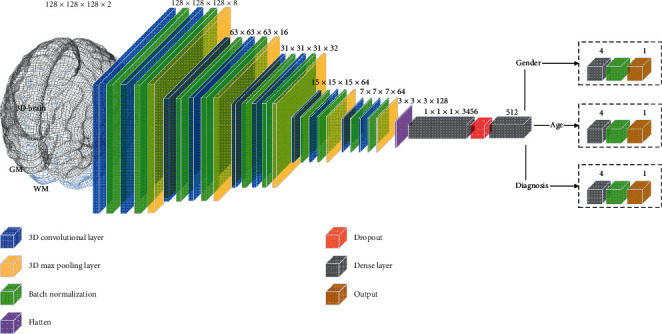
3D convolutional neural network multitask learning model. The processing steps through the layers allow the extraction of increasingly complex brain features. While batch normalization allows faster network convergence, dropout plays an important role in increasing generalization. Due to the mechanisms of multitask learning architecture, such as data amplification and attribute selection, the shared features allow faster convergence and better generalization.

The architecture of our model was designed to receive the previously transformed 3D brains as input for the neural network training. The input for training is a 5D matrix (composed of the number of examples in batch, voxel *X*-axis, voxel *Y*-axis, voxel *Z*-axis, brain tissues), where the brain tissue is a two-channel dimension composed of GM and WM. We considered only GM and WM to ensure that our models' predictions resulted from patterns directly related to differences in neurodevelopment. Therefore, the cerebrospinal fluid, the skull, and all the tissues outside the brain were discarded. That was also the reason why we did not use the complete unsegmented images. Moreover, we opted for feeding data through different channels to the model so that it had a facilitation signal to differentiate the patterns of GM (mostly neuronal nuclei) and WM (mostly axon bundles). As shown in Figure 2, the common model's body is composed of a sequence of interleaved layers of 3D convolution, batch normalization, and 3D max pooling, followed by dense and dropout layers. After the common model's body, we derived three output blocks, each composed of its own dense, batch normalization, and output layer. The output blocks are accountable to, respectively, predict gender, age, and psychiatric disorder (i.e., HC, ASD, or ADHD).

Inspired by the VGG16 network [33], we chose the ReLU activation function to provide nonlinearity [34] and used convolutional layers with receptive fields of 3 × 3 × 3 pixels and max-pooling layers with 3 × 3 × 3 pixel window and stride of 2 × 2 × 2. To improve the network convergence, we added batch normalization [35] before convolutional and dense layers. To face overfitting problems, we included *L*2 kernel regularizers (with a coefficient equal to 1 × 10^−3^) in all convolutional and dense layers and added a dropout [36] with a dropout rate of 0.5 right after the flattening of the last convolutional layer.

The loss chosen as the objective function to be minimized is expressed by the weighted sum of the loss of each output, where we opted for the Mean Squared Error for the age output and Binary Cross-Entropy for gender and diagnosis outputs. The loss weights (*W*_1_, *W*_2,_ and *W*_3_) were not tuned, remaining in the default values of the TensorFlow library (i.e., equal to 1). As the classification and regression tasks have different loss scales, the loss will be higher to the age estimation than to the classification tasks. That is, the training will tend to optimize more in the direction of the age estimation than in that of the classification tasks.

(1) $$\begin{array}{c} {\text{objective}}_{\text{loss}}=W_{1}*\text{mean}\_\text{squared}\_\text{error }\left(y_{\text{age}},\hat{y}{\;}_{\text{age}}\right),\\ +\;W_{2}*\text{binary}\_\text{crossentropy }\left(y_{\text{gender}},\;{\hat{y}}_{\text{gender}}\right),\\ +\;W_{3}*\text{binary}\_\text{crossentropy }\left(y_{\text{diagnosis}},\;{\hat{y}}_{\text{diagnosis}}\right). \end{array}$$

Our motivation for choosing a multitask learning architecture is the advantages produced by the learned features in the shared layers that are favored from the mechanisms of data amplification, attribute selection, eavesdropping, and representation bias [37]. In brief, this approach allows faster convergence and better generalization due to the extra information provided by the training signals of the related tasks [37].

### 2.5. Model Tuning and Training

Despite our preferences for using an automated method for the tuning process (e.g., grid search or Bayesian optimization), which was already employed in other works [14, 15], the hundreds of hyperparameters combinations and the long time consumed by each training session made this strategy unfeasible. Instead, the tuning was carried out based on previous knowledge and mainly insights from the publications of the VGG16 network [33], batch normalization [35], and dropout [36].

To make better use of processing time and memory resources, we set the TensorFlow mixed-precision configuration to employ both 16-bit and 32-bit floating-point types during the training phase (https://www.tensorflow.org/guide/mixed_precision). We also padded and trimmed the brain input matrix, which originally had the size of 121 × 145 × 121 to 128 × 128 × 128. This step only affected background voxels (outside the brain) whose values were all equal to −1. This procedure followed the TensorFlow performance guide, which states that feature matrices multiples of 8 or 128 should be used for best memory usage (https://cloud.google.com/tpu/docs/performance-guide).

To optimize the objective loss, we opted for a gradient-based method with adaptive learning rates named Adam [38]. The initial Adam's learning rate was set to 1 × 10^−3^, and the exponential decay rates for the first and second estimate moments were, respectively, set to 0.9 and 0.999. The loss weights from the objective function were not tuned and may be further explored in an upcoming study.

For the training sessions, the batch size was set to 32 examples, which is the maximum size that fitted in memory. As our model deals with distinct target variables with different data distributions at the same time (i.e., age, gender, and mental health status), we opted to do not balance the classes at the batch level. Thus, the examples were just randomly shuffled before the batch splits. The number of epochs was set to 1000, and a custom early stopping technique was implemented to stop the training process every time there was no improvement of at least one of the output losses in the validation set for 75 consecutive epochs. Following this strategy, most (75%) training sessions ended after running from 150 to 300 epochs. Additionally, we employed a technique called model checkpoint. Therefore, at the end of each epoch, the model was evaluated against the validation set, and the best-performing model parameters for each task were saved. This strategy provides three model versions at the end of each training session: one performing better to predict gender, another performing better to predict age, and the last performing better to predict psychiatric disorder.

At a first glance, one may argue that it is counterintuitive to save different model versions from the same multitask learning based model. However, we found in our preliminary tests that this schema reduced the models' training until convergence by three times, when compared to the time spent to train three different single-task models. Additionally, this approach helped (1) to prevent overfitting, by saving the model weights at the optimum training point, and (2) to generate model versions trained to best extract the relevant features for its main task. We used the lowest loss of each output (i.e., *Mean Squared Error* for age prediction and *Binary Cross-Entropy* for gender and psychiatric disorder predictions) as the metrics to automatically save the best checkpoints.

### 2.6. Test Procedure

Each data set (ABIDE-II and ADHD-200) was stratified (i.e., balanced) by mental health status (i.e., HC, ASD, and ADHD), randomly shuffled, and split in a 10-fold cross-validation custom scheme. Accordingly, data is initially split into 10 partitions and, in every training round, 1 partition is chosen for the test set. Then, from the 9 remaining partitions, the first 8 are assigned to the training set and the last 1 is assigned to the validation set (see Figure S1 in the Supplementary Materials). This cross-validation scheme resulted in 10 training rounds for each data set. For each round, the corresponding training set was used to train the network. The remaining validation set was employed to automatically save the best-performing models through the previously described model checkpoint technique. The test sets were kept untouched until the models were fully trained so that the performance of the final models could be assessed on an unbiased and unexplored data set. This custom validation scheme takes advantage of the robustness of a nested (double) cross-validation while preserving the lower time consumption of a nonnested cross-validation scheme.

For each training round of the 10-fold cross-validation, we obtained three final trained models: (1) optimized for gender, (2) optimized for age, and (3) optimized for psychiatric disorder classification. These models were evaluated as follows:

- All models trained with ABIDE-II data were evaluated on their corresponding test set
- All models trained with ADHD-200 data were evaluated on their corresponding test set
- The best-performing model trained with ABIDE-II data to predict age was evaluated across the full ADHD-200 data set
- The best-performing model trained with ABIDE-II data to predict gender was evaluated across the full ADHD-200 data set
- The best-performing model trained with ADHD-200 to predict age was evaluated across the full ABIDE-II data set
- The best-performing model trained with ADHD-200 to predict gender was evaluated across the full ABIDE-II data set

The chosen metrics to evaluate the models' performance in the regression task of predicting age were *MAE (mean absolute error)*, *Pearson's correlation*, *P value* of the Pearson's correlation, and *R2-score* (also known as prediction *R*^2^, cross-validation *R*^2^ or *q*^2^, which best assesses numerical accuracy for regression tasks [39]). For the tasks of predicting gender and psychiatric disorder, we used *precision* (specificity measure), *recall* (sensibility measure), *F1-score* (harmonic mean between precision and recall), and *AUC-ROC (area under the receiver operating characteristic curve)*. The *F1-score* was chosen (instead of the simple accuracy) due to its capability to evaluate unbalanced data better.

The use of unbalanced data for the gender and mental health status classifications can bias the models towards classifying minority cases as majorities [40]. To address this issue, we employed a ROC operating point selection that maximizes the harmonic mean between sensitivity and specificity [40]. That is, for each trained model, we use the validation data to find the cutoff value that best maximizes the balance between sensitivity and specificity. The chosen cutoff value is then used to collect the metrics from the test data.

### 2.7. Model Interpretability

In general, artificial neural networks have been known for their low interpretability level, sometimes being labeled as a “black box” providing little or no insight into the nature of data [14, 15]. The explanation of image-based artificial neural networks remains a challenge in the healthcare domain. To address this issue, we employed an algorithm called SmoothGrad [21]. It produces a sensitivity map of the voxels that most contribute to the neural network decisions by measuring the impact that small perturbations applied to input images produce in the output gradients. Although SmoothGrad uses the same basic methodology as other algorithms, it has the advantage of producing sharpen results due to the strategy of applying different perturbations to the same input image. Moreover, it averages the resulting maps, producing a better smoothing effect [21]. The present study employed the SmoothGrad algorithm through the open-source library implementation called tf-keras-vis (available at https://pypi.org/project/tf-keras-vis).

As quoted in the original paper [21], the sensitivity map algorithms often produce signed values. Therefore, there is considerable ambiguity in how to convert these signed values to visualization colors, as the direction of the gradient is context-dependent. To solve this ambiguity, we opted for using the absolute values of the gradients, which has the potential of producing clearer pictures [41] and was also proposed by SmoothGrad authors [21]. During the attention maps generation, the noise level was set to 20%, and the number of samples (sample size) for each input image was set to 5. Although the SmoothGrad paper shows increasing definition in the produced maps as the sample size is incremented, the processing time for this task is directly proportional to the sample size. Therefore, higher sample size values proved to be unfeasible given our limited hardware resources. Furthermore, we verified in a preliminary test that setting sample size to 10 produced the same top ROIs as setting the chosen configuration of 5. As our models have three outputs, we had to set to zero all outputs that were not the ones chosen for measurement (e.g., while generating the age sensitivity map, we set the gender and psychiatric disorder outputs to zero).

Attention maps were generated for the final models of each of the 10 cross-validation folds from their corresponding test set. These maps were first averaged from their test set examples and then were normalized and averaged across all the 10 training rounds, resulting in an attention map for each task (i.e., predicting age, gender, or psychiatric disorder) and for each data set (ABIDE-II and ADHD-200). This strategy allowed for capturing common structural brain regions that are most descriptive for the models' decision-making in each task.

As the final generated maps have the same 3D shape of the input images (localized in the MNI space), we could identify the most predictive brain ROIs taking the intersection between the attention maps and the AAL3 3D brain atlas [22]. Finally, the maps were rendered in the MRIcron viewer (https://www.nitrc.org/projects/mricron) to provide more interpretable brain visualizations.

### 2.8. Experiments Setup

The sMRI processing steps were done through the software SPM12 v7771, *Python* v3.6.9, and TensorFlow v2.1.0, running on a local Linux desktop (CPU 3.2 GHz Octa Core, 32 GB ram). After the sMRI processing, the TFRecord files were uploaded to a Google Cloud storage bucket.

Our machine learning experiments were conducted using a Google Colab instance (https://colab.research.google.com/): CPU 2.3 GHz Dual Core, 12 GB ram, attached to a Cloud TPU v2 (180 teraflops/s speed and 64 GB ram), connected to the aforementioned storage bucket, Trough *Python* v3.6.9, and TensorFlow v2.3.

## 3. Results

The training and testing phases occurred successfully with adequate processing time for all models. Output metrics collected showed that CNN3D models were able to learn and predict age and gender with a high confidence level in both ABIDE-II (MAE = 1.63 ± 0.28, AUC = 0.82 ± 0.06) and ADHD-200 (MAE = 1.43 ± 0.22, AUC = 0.85 ± 0.04) data sets. For both age and gender predictions, models trained on ADHD-200 data had slightly higher performance than those trained on ABIDE-II, including when we evaluated the best-performing cross-validation models from one data set across the other distinct full data set (MAE = 1.57, AUC = 0.89 vs MAE = 1.64, AUC = 0.79).

**Figure 3:**
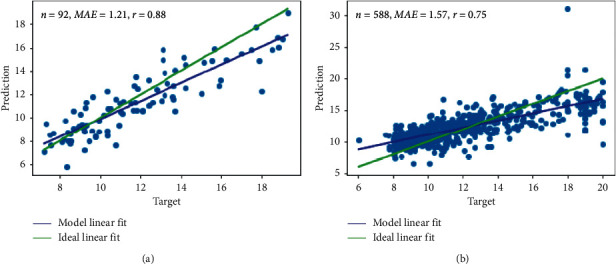
Scatter plots between predicted and target ages. (a) Ages prediction on the test set from the best-performing model of ADHD-200 10-fold cross-validation. (b) The same best-performing model, which was trained with ADHD-200 data, evaluated across the full ABIDE-II data set. Note. *r*: Pearson's correlation between predicted and target ages, MAE: the mean absolute error of the predictions, and *n*: the evaluated sample size.

For the age prediction, the ADHD-200 models evaluated in a 10-fold cross-validation scheme on the test set obtained an MAE (mean absolute error) of 1.43 years, reaching a mean Pearson correlation of 0.84 between the correct targets and the models' predictions and a mean R2-score (also known as prediction *R*^2^, cross-validation *R*^2^ or *q*^2^) of 0.62. The best-performing model of the aforementioned cross-validation, which was trained with ADHD-200 data, achieved an MAE of 1.21 years on its corresponding test set, and when evaluated across the full ABIDE-II data set, it reached an MAE of 1.57 years and a Pearson correlation of 0.75 between targets and predictions (see Figure 3).

For gender prediction, the ADHD-200 models evaluated in a 10-fold cross-validation scheme on the test set obtained a mean AUC-ROC of 0.85, with precision = 0.84, recall = 0.81, and F1-score = 0.83. The best-performing model of the above-mentioned cross-validation, which was trained with ADHD-200 data, achieved an AUC-ROC of 0.91 on its corresponding test set, and when evaluated across the full ABIDE-II data set, it achieved an AUC-ROC of 0.89, with precision = 0.90, recall = 0.87, and F1-score = 0.89 (see Figure S2 in the Supplementary Materials).

For psychiatric disorder classifications, the models had poor learning, performing close to the random guessing. The ADHD-200 models evaluated in 10-fold cross-validation on the test set obtained a slightly better performance predicting ADHD (AUC-ROC = 0.61), while the models trained on ABIDE-II to predict ASD obtained a mean AUC-ROC = 0.54. All the evaluated metrics are presented in Table 2.

To access the statistical impact of the total brain volume on estimations, we calculated the AUC-ROC and Person's correlation (*r*), respectively, to gender and age concerning the sum of brain voxels from each subject. Thus, the ABIDE-II data (*N* = 588) yielded AUC-ROC = 0.76 and *r* = 0.03, while the ADHD-200 data (*N* = 922) resulted in AUC-ROC = 0.79 and *r* < 0.001. These results show that total brain volume is not related to age, while it may have influenced gender estimations. However, the focus of our work is the study of neurodevelopment, which is assessed mainly through age estimations.

The top 10 most representative ROIs from ADHD-200 models to classify gender are cingulate posterior gyrus (left and right), anteroventral thalamus (left and right), lateral posterior thalamus (right), mediodorsal lateral thalamus (right), mediodorsal medial thalamus (left and right), ventral anterior thalamus (right), and ventral lateral thalamus (right). In the ABIDE-II sample, the top 10 most representative ROIs comprised calcarine fissure (right), cingulate posterior gyrus (right), cerebellum lobe III (left), lingual gyrus (right), rolandic operculum (left), substantia nigra pars reticulata (left), pulvinar lateral thalamus (right), pulvinar medial thalamus (right), and vermis (lobes III and IV-V). The cingulate posterior gyrus (right) emerged as a top ROI on both ADHD-200 and ABIDE-II models for gender prediction.

Among age prediction models, the substantia nigra pars reticulata (left) arose in the top ROIs of both ADHD-200 and ABIDE-II models. ADHD-200 models retrieved the following regions as the top 10 ROIs: cingulate posterior gyrus (right), precentral gyrus (right), rolandic operculum (right), globus pallidus (left), substantia nigra pars reticulata (left), intralaminar thalamus (left), lateral geniculate thalamus (left), medial geniculate thalamus (left), pulvinar lateral thalamus (left), and vermis (lobes IV-V). ABIDE-II models top 10 focused ROIs comprised the following regions: the amygdala (right), middle cingulate (right), olfactory cortex (right), paracentral lobule (right), ventral tegmental area (right), vermis (lobes III and X), substantia nigra pars compacta (right), and substantia nigra pars reticulata (left and right). Interestingly, the vermis lobe III arose as a focused top 10 prediction ROI for gender and age in ABIDE-II models, and the vermis lobes IV-V emerged for both gender and age predictions in both samples. A compilation of the top-focused ROIs is depicted in Figure S3 in the Supplementary Materials.

**Figure 4:**
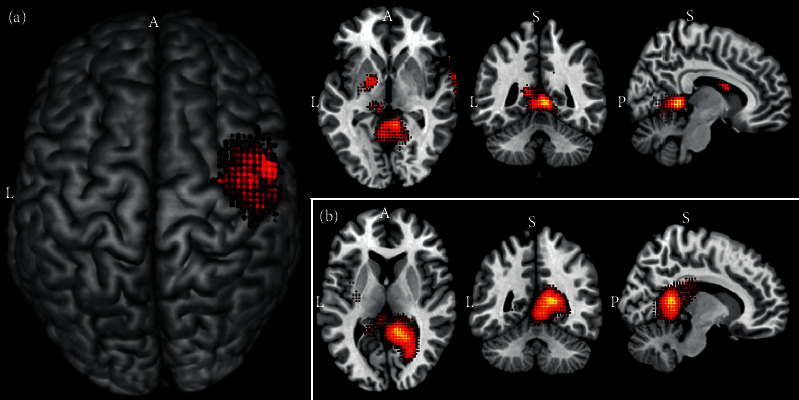
Top ROIs from gradients' attention maps perspective. (a) Top regions to predict the age by averaged attention of 10-fold ADHD-200 models. (b) Top regions to predict the gender by averaged attention of 10-fold ABIDE-II models. L: left, A: anterior, P: posterior, S: superior.

As previously explained, model interpretability of artificial neural networks is sometimes challenging, which limits its applicability in clinical scenarios. Therefore, these models are deemed to be “black box,” with little practical impact. However, we implemented a visualization approach to add to the models' interpretability. In Figure 4, we present an implementation of this procedure by adding the averaged gradients' attention maps as an overlayed layer of an MRIcron's brain template. It shows a practical example of visual outputs from artificial neural networks, where the top 10 predictive ROIs from gradients attention maps were accurately plotted in a clinically relevant representation of the brain.

## 4. Discussion

In this study, we transformed brain sMRI of youth via VBM, from large and heterogeneous data sets, and used the resultant GM and WM as input for training 3D's convolutional neural network with multitask learning models to predict age, gender, and psychiatric disorder. Then, the resultant trained models were used to map the top representative ROIs for the tasks of predicting age and gender. To achieve consistency and avoid biased results, we used a set of methods in line with the literature's best practices.

The ADHD-200-trained models had a slightly better performance than the models trained with ABIDE-II data, possibly because the first data set has higher homogeneity in data than the second [12]. The cross-data set evaluation proved the models' generalization capability to predict age and gender with high confidence even in unknown data sets with distinct confounding variables such as type of psychiatric disorder, scanner acquisition parameters, and subjects' distribution of age and gender.

To the best of our knowledge, the performance of our approach is in line with the state-of-the-art in brain aging detection, achieving an MAE = 1.43 years in 10-fold cross-validation on the test set. A study of Wang and coworkers [42] reached an MAE = 1.38 years in a subset from ADHD-200 with a similar age range to ours; however, their results were only based on healthy individuals, and their approach employed handcrafted feature extraction and selection based mainly on cortical thickness and curvatures. Another study, by Franke and colleagues [43], achieved an impressive MAE = 1.1 years in one of their test partitions and an MAE = 1.22 years from the averaged performance from all six test partitions. Unlike our work, Frank and coworkers employed a data set [44] acquired using a unified set of scanner parameters, from healthy subjects only, after rigorous filtering for dozens of confounding factors that could influence the healthy brain maturation during childhood and adolescence (i.e., individuals with preterm birth, alcohol or drug abuse during the gestational period, low IQ, and dozens of other confounding factors were excluded). Greater data uniformity, combined with smaller sample sizes, than that employed by us possibly provided good conditions so that both studies could achieve high accuracy [42,43], although it may have occurred at the cost of generalizability [12]. Different from our approach, these studies [42,43] employed a machine learning algorithm called relevance vector machine (RVM) [45], which is a Bayesian alternative to support vector machine. Therefore, RVM has the advantage of requiring less computational power than CNN3D.

Another study employed a CNN3D to predict age from brain sMRI in raw format versus sMRI processed by VBM. Cole and colleagues [46] achieved slightly better performance when they used VBM (MAE = 4.16 years) in comparison to raw sMRI (MAE = 4.66 years). However, they have only evaluated healthy subjects, with ages ranging from 18 to 90 years of age. Therefore, these differences do not allow a direct comparison of the model performance to our work. Additionally, unlike our study, Cole and coworkers [46] did not assess brain biomarkers (ROIs) from their model's predictions.

Although our approach presented a high capability to learn how to estimate age and gender, it did not perform well in classifying psychiatric disorders, achieving modest AUC-ROC and F1-score metrics when differentiating between HC, ASD, and ADHD. Therefore, the results show that our models were close to the random guessing for these tasks. Possibly, the underlying structural alterations from these conditions are subtle enough so that they are not efficiently detectable by CNN3D trained with sMRI from large and heterogeneous data sets. In psychiatric disorders, large and heterogeneous data samples tend to deliver high confidence and generalization power. However, at the same time, they tend to lead to low accuracies, which is an important limitation that possibly has also affected our main results [12]. Another source for investigation, in future work, is to evaluate the effect of tuning the weights from the objective loss function to prioritize the mental health status classification. The dynamic task prioritization for multitask learning [47] seems to be an interesting approach for this goal. This method proposes the dynamic adjustment of loss weights across the training process to prioritize the most difficult tasks.

The brain ROIs we identified (see Results) as being most representative for gender and age detection come in line with several distinct studies that reported these regions as being related to differentiation of gender, aging, or both [48–54].

For gender, Witte and coworkers [48] used Statistical Parametric Mapping to calculate GM volume differences between men and women, and among other statistically significant findings, they discovered that men had more GM than women in vermis, cerebellum, and right calcarine, while women had more GM than men in the lingual gyrus. Another study, by Menzler and colleagues [49], employed diffusion tensor MRI to discover microstructural differences between genders in the WM of the thalamus; Menzler and coworkers [49] also found differences in the cingulum confirming previous works, suggested that their findings were due to differences in myelination or glial cell morphometry, and stated that previous functional MRI studies found gender differences in thalamic activation during the processing of emotional stimuli or unpleasant linguistic information. Recent findings suggest that not only gender but also pubertal status may influence brain development [55]. Thus, the role of these features can be a source of further exploration in future work.

For age-related ROIs, Tomasi and Volkow [53] used functional MRI to evaluate the functional connectivity density (FCD) of networks concerning brain aging of healthy subjects and found that a long-range FCD in the default-mode network (DMN), which includes the posterior cingulate, decreased with age, while FCD in other two subcortical networks including thalamus and amygdala increased with age; more recently, an improved neuroanatomical model of DMN [56] not only included amygdala and thalamus in DMN but found that the thalamus has a centrality role in DMN. Another study used functional MRI [54] to find that in children the ventral tegmental area had lower connectivity to the amygdala and higher ventral tegmental area connectivity to the thalamus, globus pallidus, and vermis than in adults; this study [54] also revealed that in children the substantia nigra had higher connectivity to the amygdala, globus pallidus, and thalamus than in adults, and similarly the connectivity of language areas (including rolandic operculum) and middle cingulate was weaker with the ventral tegmental area than with substantia nigra for adults.

Taking it collectively, the morphological changes detected by our models and confirmed in other studies [48–54] are possibly related to the highly coordinated and sequenced events characterized by both progressive (myelination) and regressive (synaptic pruning) processes, which alter WM and GM volumes with different patterns for each gender, and are most dynamic from childhood to early adulthood [57].

These findings reinforce our hypothesis that CNN3D is able to detect complex brain morphological features, previously detectable by high-resolution diffusion tensor MRI and by functional MRI. Following Pinaya [15], while the standard mass-univariate techniques consider each brain structure as an independent unit, multivariate methods (such as the one we used) may be additionally based on interregional correlations leading individual regions to present high discriminative power due to two possible reasons: (a) a difference in volume/thickness between groups in that region; (b) a difference in the correlation between that region and other areas between groups. Therefore, discriminative brain networks are best interpreted as a spatially distributed pattern rather than as individual regions.

As our multitask learning architecture is optimized to perform all tasks at the same time (i.e., predicting gender, age, and psychiatric disorder), the learning process in the common model's body may favor the extraction of the brain features that are relevant to more than one task. On the other hand, each specific output block is exclusively optimized, selecting only the appropriate set of features that best help to accomplish its unique individual task.

Due to the complexity arising from the nonlinearity of artificial neural networks, our methods do not allow mapping the differences inside ROIs that are relevant to the models' decisions, that is, which patterns of increase/decrease in cortical volume of focused ROIs are accountable for a given model decision. Another limitation of the current study is that it does not explain the obtained performance results, that is, which methods are accountable for which performance improvements. Therefore, this topic is still open and can be further explored in future work.

Our approach was not sufficient to adequately classify ASD and ADHD. In contrast, the performance and generalization power achieved in predicting age (i.e., neurodevelopment) can pave the way for future work through the indirect estimation of psychiatric disorders. By training our model to predict the age of healthy individuals only (to be done), psychiatric conditions can be estimated by calculating the difference between the brain's predicted age and the individual's chronological age [46]. Increased brain predicted age has been detected in individuals progressing to Alzheimer's, in schizophrenia, in epilepsy, and Down's syndrome [58–61]. At the same time, decreased brain predicted age has been used to highlight the protective influences exerted by meditation, by increase in education levels, and by physical exercises [62, 63].

## 5. Conclusions

In conclusion, this study proved the ability of CNN3D models trained with GM and WM, processed via VBM, to accurately estimate age (i.e., neurodevelopment) and gender. Therefore, the achieved results endorse the hypothesis that our approach is able to detect complex brain patterns. Although the models were not able to efficiently differentiate between HC, ASD, and ADHD, the high performance and generalization power achieved in age estimation can pave the way for future work, through the indirect estimation of psychiatric disorders. The strategy of generating 3D brain saliency maps via SmoothGrad [21] and intersecting the results with the 3D AAL3 brain atlas [22] was successfully achieved. Therefore, it provided clinically relevant identification of most representative biomarkers (ROIs) during models' decisions and proved to be a viable alternative to deal with the well-known low interpretability problem of deep learning models. Finally, the results achieved by the presented approach reinforce the hypothesis that it can be successfully adapted to tackle a varying set of problems involving brain morphological alterations.

## Figures and Tables

**Table 1:** Subjects' demographic and phenotypic information.

Data set	*N*	Male (%)	Female (%)	Age, *y* ± SD	Age range, *y*	HC (%)	ASD (%)	ADHD (%)
ABIDE-II	580	73.8	26.2	12.12 ± 3.16	6.0–20.0	56.7	43.3%	—
ADHD-200	922	63.1	36.9	11.72 ± 2.99	7.1–19.9	61.3	—	38.7%

The number of subjects (N) is shown in numbers, while age is in years ± standard deviation and in range of minimum–maximum years of age.

**Table 2:** Performance metrics of the test procedure.

*Regression models*	*n*	*MAE, y*	*r*	*Pvalue*	*R2-scr*
Age: ABIDE-II 10-fold CV on test set	58	1.63 ± 0.28	0.76 ± 0.07	<0.001	0.54 ± 0.1
Age: ABIDE-II model on ADHD-200 full data	922	1.64	0.72	<0.001	0.50
Age: ADHD-200 10-fold CV on test set	92	**1.43** ± 0.22	0.84 ± 0.04	<0.001	0.62 ± 0.08
Age: ADHD-200 model on ABIDE-II full data	580	**1.57**	0.75	<0.001	0.56

Classification models	*n*	*Precision*	*Recall*	*F1-scr*	*AUC-ROC*
Gender: ABIDE-II, 10-fold CV on test set	58	0.87 ± 0.06	0.80 ± 0.08	0.83 ± 0.04	0.82 ± 0.06
Gender: ABIDE-II model on ADHD-200 full data	922	0.76	0.80	0.78	0.79
Gender: ADHD-200, 10-fold CV on test set	92	0.84 ± 0.03	0.81 ± 0.06	0.83 ± 0.03	**0.85** ± 0.04
Gender: ADHD-200 model on ABIDE-II full data	580	0.90	0.87	0.89	**0.89**
ASD: ABIDE-II, 10-fold CV on test set	58	0.46 ± 0.04	0.70 ± 0.18	0.55 ± 0.06	0.54 ± 0.06
ADHD: ADHD-200, 10-fold CV on test set	92	0.48 ± 0.07	0.55 ± 0.20	0.50 ± 0.11	**0.61** ± 0.07

The performance indicators from 10-fold cross-validation are presented in their averaged values ± standard deviation. The chosen model for the cross-data set evaluation is the best-performing model of 10-fold cross-validation. For the column titles, *r* is the Pearson's correlation between predicted and target ages, *n* is the sample size, and R2-scr is the prediction *R*^2^ (also known as cross-validation *R*^2^ or *q*^2^). Values in bold are metrics of the best-performing trained models. ASD: autism spectrum disorder; ADHD: attention deficit hyperactivity disorder.

## Data Availability

The data used in this study were obtained from two public data sets: Autism Brain Imaging Data Exchange II (ABIDE-II) and Attention Deficit Hyperactivity Disorder (ADHD-200). Both data sets can be downloaded from the NeuroImaging Tools & Resources Collaboratory Image Repository (NITRC-IR: https://www.nitrc.org/ir/). The data were collected and made publicly available according to the responsibility and approval of the given local ethics by each project. Detailed information for these data sets and their acquisition parameters can be retrieved from ABIDE-II (http://fcon_1000.projects.nitrc.org/indi/abide/abide_II.html) and ADHD-200 (http://fcon_1000.projects.nitrc.org/indi/adhd200/).

## Supplementary Materials

Supplementary Materials Figure S1 schematically demonstrates the adopted custom validation scheme, which takes advantage of the robustness of a nested cross-validation while preserving lower time consumption. Figure S2 displays the confusion matrices from the best-performing ADHD-200 model classifying gender on its test set and across the full ABIDE-II data set. Figure S3 presents the most representative brain regions to estimate age and gender from ADHD-200- and ABIDE-II-trained models.
