# Supplementary material for: Estimating Gender and Age from Brain Structural MRI of Children and Adolescents: A 3D Convolutional Neural Network Multitask Learning Model
Source: Comput Intell Neurosci. 2021 May 26;2021:5550914. doi: 10.1155/2021/5550914 (PMC8172319; doi:10.1155/2021/5550914)
Supplement: Supplementary Materials — Figure S1 schematically demonstrates the adopted custom validation scheme, which takes advantage of the robustness of a nested cross-validation while preserving lower time consumption. Figure S2 displays the confusion matrices from the best-performing ADHD-200 model classifying gender on its test set and across the full ABIDE-II data set. Figure S3 presents the most representative brain regions to estimate age and gender from ADHD-200- and ABIDE-II-trained models. [file 5550914.f1.docx]

**Supplementary Material**

Figure S1: A 10-fold cross-validation custom scheme takes advantage of the robustness of a nested (double) cross-validation while preserving the lower time consumption of a non-nested cross-validation scheme. The validation set is employed to evaluate specific metrics and save the best performing models through the model checkpoint technique. The test set remains untouched until the models are fully trained, allowing the performance of the final models to be assessed on unbiased and unexplored data.


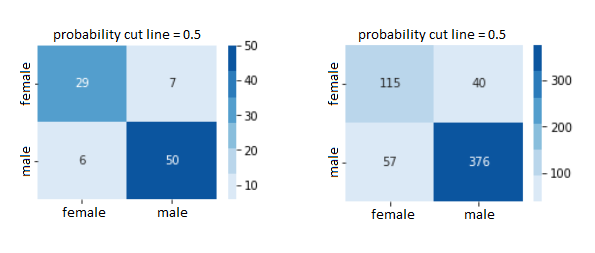


P R E D I C T I O N

P R E D I C T I O N

T A R G E T

T A R G E T

**a**

**b**

Figure S2: Confusion matrices of predicted and target genders. (a) Gender prediction on the test set from the best-performing model of the ADHD-200 10-Fold cross-validation. (b) The same best-performing model, which was trained with ADHD-200 data, evaluated across the full ABIDE-II data set.

GENDER PREDICTION ROIs

AGE PREDICTION ROIs

Figure S3: Top regions of interest to predict gender and age. Acronyms: L = left, R = right, 1 = ADHD-200 trained models, 2 = ABIDE-II trained models
